# First Detection of the Larval Chalkbrood Disease Pathogen *Ascosphaera apis* (Ascomycota: Eurotiomycetes: Ascosphaerales) in Adult Bumble Bees

**DOI:** 10.1371/journal.pone.0124868

**Published:** 2015-04-17

**Authors:** Sarah A. Maxfield-Taylor, Alija B. Mujic, Sujaya Rao

**Affiliations:** 1 Department of Crop and Soil Science, Oregon State University, Corvallis, Oregon, United States of America; 2 Department of Botany and Plant Pathology, Oregon State University, Corvallis, Oregon, United States of America; University of California-San Diego, UNITED STATES

## Abstract

Fungi in the genus *Ascosphaera* (Ascomycota: Eurotiomycetes: Ascosphaerales) cause chalkbrood disease in larvae of bees. Here, we report the first-ever detection of the fungus in adult bumble bees that were raised in captivity for studies on colony development. Wild queens of *Bombus griseocollis*, *B*. *nevadensis and B*. *vosnesenskii* were collected and maintained for establishment of nests. Queens that died during rearing or that did not lay eggs within one month of capture were dissected, and tissues were examined microscopically for the presence of pathogens. Filamentous fungi that were detected were plated on artificial media containing broad spectrum antibiotics for isolation and identification. Based on morphological characters, the fungus was identified as *Ascosphaera apis* (Maasen ex Claussen) Olive and Spiltoir, a species that has been reported earlier only from larvae of the European honey bee, *Apis mellifera*, the Asian honey bee, *Apis cerana*, and the carpenter bee *Xylocopa californica arizonensis*. The identity of the fungus was confirmed using molecular markers and phylogenetic analysis. *Ascosphaera apis* was detected in queens of all three bumble bee species examined. Of 150 queens dissected, 12 (8%) contained vegetative and reproductive stages of the fungus. Both fungal stages were also detected in two workers collected from colonies with *Ascosphaera*-infected *B*. *nevadensis* queens. In this study, wild bees could have been infected prior to capture for rearing, or, the *A*. *apis* infection could have originated via contaminated European honey bee pollen fed to the bumble bees in captivity. Thus, the discovery of *A*. *apis* in adult bumble bees in the current study has important implications for commercial production of bumble bee colonies and highlights potential risks to native bees via pathogen spillover from infected bees and infected pollen.

## Introduction

The fungus *Ascosphaera* (Ascomycota: Eurotiomycetes: Ascosphaerales) is primarily associated with larvae of bees and bee products [1,2]. There are 28 known species worldwide, the majority of which are saprotrophs on pollen stores, honey, larval feces, and nesting material [3]. Some species are pathogenic and cause chalkbrood disease in larvae of social bees and solitary bees [1, 2]. These include *A*. *aggregata* Skou, *A*. *apis* (Maassen ex Claussen) Olive et Spiltoir, *A*. *atra* Skou et Hackett, *A*. *major* (Prokschl et Zobl) Skou and *A*. *proliperda* Skou [2, 4, 5, 6]. Bee hosts of *Ascosphaera* include the European honey bee, *Apis mellifera* L. (Apidae), leaf cutting bees, *Megachile* spp., mason bees, *Osmia* spp. (Megachilidae), and sweat bees, *Nomia* spp. (Halictidae) [2, 7, 8]. In isolated instances, *Ascosphaera* growth has been reported from larvae of the bumble bee *Bombus terrestris* L. (Apidae) [9], larval feces of a dipteran [2] and from grass silage [10]. The fungus has, however, never been reported from any adult bee or other adult insect.

Infections of *Ascosphaera* and other pathogenic members of the Ascosphaeraceae occur through the gut rather than externally through the cuticle [3]. The spores germinate in the anaerobic environment of the hindgut, and mycelia that are produced reach the abdomen where they develop aerobically before they penetrate the cuticle [11]. Initially, infected larvae become spongy and white but, as the infection progresses, the larvae harden and become chalk-like. *Ascosphaera* produces unique fruiting bodies comprised of spore balls held within a double walled spore cyst, called cleistothecia. These develop on the cuticle and turn the larvae greenish-brown, grey, or black. An exception to this is *A*. *aggregata*, which forms cleistothecia directly under the larval cuticle [4].

The impact of *Ascosphaera* on bees varies with the bee host species. Infections of *A*. *apis* in European honey bee colonies are rarely treated [1] while infections of *A*. *aggregata* in the alfalfa leafcutting bee, *Megachile rotundata* (F.) have considerable economic impact as the pathogen causes the devastating chalkbrood disease in larvae of this bee species. The alfalfa leafcutting bee is raised commercially for pollination of seed crops of alfalfa (*Medicago sativa* L.), and treatment of *A*. *aggregata* is usually required [12, 13]. An unidentified *Ascosphaera* sp. was reported from laboratory-reared larvae of the bumble bee, *B*. *terrestris* by Přidal et al. [9] but in a follow up study [14] the fungus was not isolated. Bumble bees have been observed to carry ascospores of *Ascosphaera*. However, the fungus has not been recorded parasitizing them in spite of the intensive research into host parasite interactions, and hence it has been believed that the fungus is unlikely to infect bumble bees [8]. Here, we document the first ever incidence of *Ascosphaera* infection in adult bumble bees. The pathogen was detected during a study that examined mortality factors of bumble bees collected from the wild and raised in captivity. The objective of this study was to determine prevalence of the fungus in bumble bee queens used for colony production, and to isolate it and identify it to species.

## Materials and Methods

Wild bumble bee queens belonging to three species, *B*. *griseocollis* (DeGeer), *B*. *nevadensis* Cresson, and *B*. *vosnesenskii* Radoszkowski were collected from agricultural fields and urban landscapes in and around the city of Corvallis (45.56° N, 123.26° W) in western Oregon on the west coast of USA. These species are not endangered or protected. The bees were collected from private lands, and no specific permission was required. Bees were collected by hand using vials, from February through May 2011, and maintained for establishment of nests under laboratory conditions following protocols described by Plowright and Jay [15] and Pomeroy and Plowright [16]. Queens and their offspring were maintained at 28°C and supplied with artificial nectar and pollen patties made from ground European honey bee pollen and ProSweet liquid sugar blend (Mann Lake Ltd., Hackensack, MN). Pollen was harvested from European honey bee hives and frozen at -20°C until use. Colonies were examined on a daily basis, and queens that died during rearing were frozen at -40°C within 24 hours for subsequent examination for the presence of pathogens. Queens that did not initiate a colony within one month of capture were also preserved and examined.

In all, 50 queens belonging to each of the three species were dissected and tissues were examined at 400X magnification using a Leica DM1000 microscope. When filamentous fungi were detected, a small sample (approximately 1mm^2^) was plated on artificial media for fungal isolation. Tissue samples from 4 workers of *B*. *nevadensis* and 1 of *B*. *vosnesenskii* from colonies of queens that died after initiating a colony were also examined. The percentage number of infected queens was calculated of each species. The Clopper-Pearson Method [17] was used to determine a 95% confidence interval for *Ascosphaera* incidence.

### Fungal isolation

All fungal cultures were isolated directly from the affected tissues and cultured at room temperature (25°C) on petri plates of Potato-Dextrose Agar (PDA) (BD Difco, Franklin Lakes, New Jersey) containing broad spectrum antibiotics (50 ppm streptomycin and ampicillin) until reproductive structures became apparent. For most cultures, reproductive structures were apparent to the naked eye within 1–4 weeks of growth. Fungal cultures of interest were selected and grown in liquid culture on Potato-Dextrose Broth (PDB). Liquid cultures were initiated by the aseptic transfer of a small piece (1cm^2^) from agar cultures into 200 ml of PDB, shaken, and incubated for up to 7 days at room temperature. At the time of fungal harvest, all liquid was removed from the samples using a Büchner flask. Fungal tissue was separated from agar residues and stored at -80°C.

### Morphological identification

Morphological characterization of fungal cultures was performed using a Leica DM1000 microscope and fungal tissue was slide mounted for microscopy in distilled water. All measurements were performed in Leica Application Suite version 3 using digital micrographs taken with a Leica DFC320 microscope camera.

### Molecular identification

DNA was extracted from fungal tissue using the fastDNA kit (MP Biomedicals, Santa Ana, CA) following manufacturer protocols. Molecular identification of fungal cultures was performed by sequencing and analysis of the internal transcribed spacer (ITS) region of the nuclear rDNA. Polymerase chain reaction (PCR) was conducted in 25 μl reactions using 1 μl of template DNA, 12.5 μl Optimization Buffer E (PCR optimization kit, Epicentre Biotechnologies, Madison, WI), 0.2 μl Genscript TAQ polymerase (Genscript, Piscataway, NJ), 7.3 μl molecular grade water, and the fungal specific primer pair ITS1F and ITS4 (2 μl each at 10 μM) [18]. PCR thermocycling conditions were as follows: Initial template denaturation at 94°C for 2 minutes, followed by 10 cycles of denaturation (94°C for 40 seconds), annealing (52°C for 45 seconds) and extension (72°C for 2:30 minutes), followed by 35 cycles of denaturation (94°C for 40 seconds), annealing (47°C for 45 seconds) and extension (72°C for 2:30 minutes), a final extension at 72°C for 2 minutes, and completed by a 4°C storage cycle until samples could be retrieved from the thermocycler. PCR products were visualized on 2% agarose using ethidium bromide and a UV transilluminator. Only those PCR products that visualized as a single distinct band under UV illumination were sequenced. PCR products were sequenced in the forward direction (ITS1F) by the Center for Genome Research and Biocomputing at Oregon State University. Sequence data was compared to the GenBank sequence database using the BLAST tool available at the website of the National Center for Biotechnology Information (NCBI) (http://blast.ncbi.nlm.nih.gov/Blast.cgi).

### Phylogenetic analysis

Two fungal cultures isolated from queens were selected for sequencing. Sequence data that showed high identity to *Ascosphaera* species in BLAST analyses were subjected to phylogenetic analysis to confirm species identity. ITS sequence data derived from fungal cultures in this study were concatenated to an ITS dataset previously used to determine infrageneric relationships in the genus *Ascosphaera* [6]. Additional ITS sequences from two voucher strains of *A*. *apis* at the American Type Culture Collection (ATCC MYA-4450, genbank accession # FJ172292; ATCC MYA-4451, genbank accession # FJ172293) and the strain used in the *A*. *apis* genome sequencing project [19] were also concatenated into the dataset. The genus *Ascosphaera* is contained within the fungal subclass Eurotiomycetidae. Hence an ITS sequence from another member of this subclass, *Aspergillus terreus* strain NIH2624, was used as an outgroup to the analysis. The ITS sequence from the genome strain of *A*. *apis* was obtained by using the BLAST search tool available through the website of the Baylor College of Medicine (https://www.hgsc.bcm.edu/arthropods/honey-bee-genome-project) to search the genome of *A*. *apis* for sequences with high sequence identity to ITS sequences derived in this study. The ITS sequence of *A*. *terreus* NIH2624 was obtained in a similar fashion using the BLAST search tool available at the AspGD website (http://www.aspergillusgenome.org/). Sequence data were aligned using the CLUSTALw algorithm as implemented in BioEdit 7.1.3.0 [20] followed by visual inspection and editing. The most appropriate model of evolution for this dataset was determined using the program jModelTest2 [21]. Phylogenetic analysis was performed using the maximum likelihood algorithm implemented in RAxML 7.2.6 [22] and Bayesian Markov-Chain Monte Carlo algorithm implemented in MyBayes 3.2.2 [23]. Both analyses were run under the GTR-gamma model of evolution. RAxML was executed using 1000 bootstrap replicates and MrBayes was run for 1000000 generations with 1000 sample points under default prior probability settings.

## Results

Both vegetative and reproductive stages of *Ascosphaera* were detected in 12 [8% (95% CI, 4–14%)] of the 150 bumble bee queens examined in the study. The fungus was present in queens of all three species. *Bombus nevadensis* queens had the highest infection [12% (95% CI, 5–24%)], followed by *B*. *vosnesenskii* [8% (95% CI, 2–19%)], while *B*. *griseocollis* had the least [4% (95% CI, 0.5–14%)]. The majority (11 out of 12) of queens that were observed to be infected had died during rearing in captivity. Death in these queens occurred 21–121 days after queens were placed in rearing boxes. However, one *B*. *nevadensis* queen that was found infected with *Ascosphaera* had been frozen before natural death. In addition, two *B*. *nevadensis* workers from different colonies with infected queens that died during rearing were observed to be heavily colonized with both vegetative and reproductive stages of *Ascosphaera*.

### Morphological description

**Fig 1:**
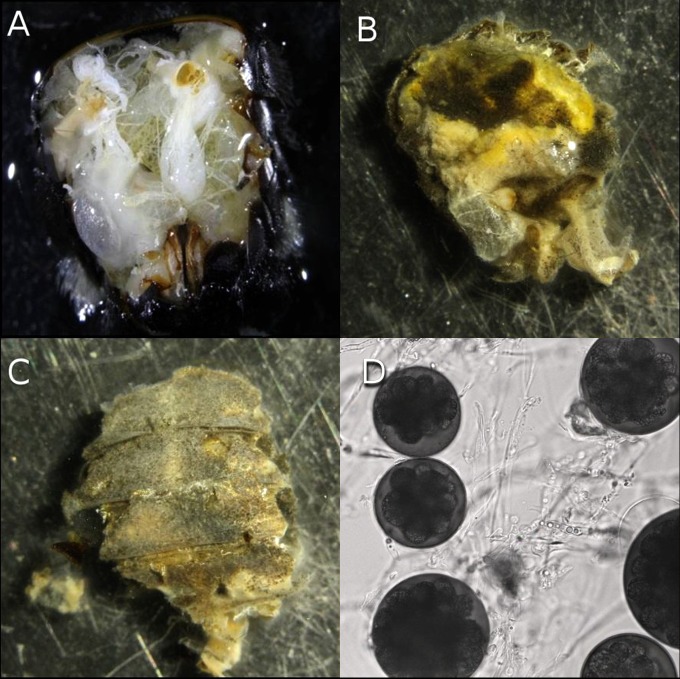
Stages of *A*. *apis* colonization of abdominal tissue of *B*. *vosnesenskii*. (A) Healthy tissues. (B) Near complete colonization with cleistothecia (darkened areas) visible. (C) Complete colonization; internal organs no longer visible. (D) Internal spore balls visible in cleistothecia (400X).

In infected adults of all three bumble bee species, the entire body cavity was filled with white spongy mycelia that were not visible externally. Bumble bee organs were unrecognizable while cleistothecia that are typical in the genus *Ascosphaera* were detected throughout the abdomen (Fig 1). Morphological characteristics of the fungus were a near perfect match for those previously described for *A*. *apis* [4]. Measurements of *A*. *apis* microscopic structures made in this study are as follows: Cleistothecia globose 34–85 μm (n = 25, average: 57.57, median: 57.09) in diameter with a thin and friable wall 1.3–1.86 μm (n = 12, average: 1.58, median: 1.6) that breaks down upon disturbance. At maturity, cleistothecia packed with globose spore masses 9–18 μm in diameter. Ascospores are hyaline and measuring 1.87 × 3.45 μm on average (n = 25, min: 1.5 × 2.88 μm, max: 2.17 × 4 μm).

### Molecular identification

The unknown *Ascosphaera* was identified as *A*. *apis* based on ITS sequence data.

### Phylogenetic analysis

**Fig 2:**
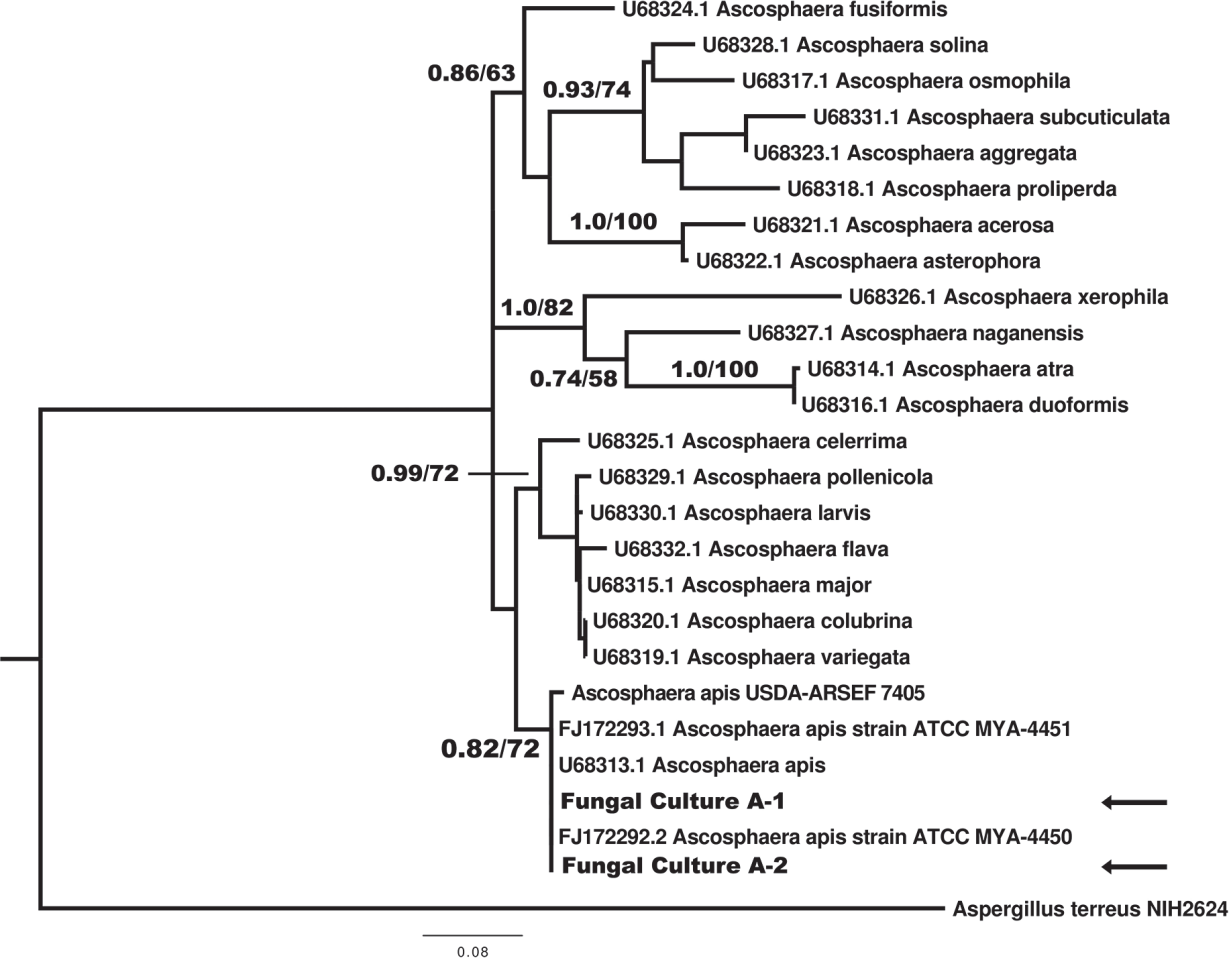
Phylogeny of the internal transcribed spacer region for selected *Ascosphaera* species. The phylogeny was inferred under both the maximum likelihood methodology in RAxML and Bayesian methodology in MrBayes using the GTRGAMMA model of evolution. 1,000 RAxML bootstrap replicates were used and MrBayes was run for 1,000,000 generations with 1000 sample points. Bayesian posterior probabilities (PP) greater than 0.70 and Bootstrap support (BS) values greater than 50 are shown for major phylogram branches (PP/BS). Genbank accession numbers precede taxon name for those sequences that were derived from Genbank. Taxa denoted in bold face as “fungal culture” represent novel sequence data from this study that are derived from cultures of *Ascosphaera apis* isolated from bumble bee queens. Fungal culture A1 has been deposited at the USDA ARSEF insect pathogen collection (culture ID ST-OR11-A1) and the ITS sequence for this culture is deposited in Genbank (accession #KJ158165). Sequences for *Aspergillus terreus* and *Ascosphaera apis* USDA-ARSEF 7405 were derived from the genome sequences available at http://www.aspgd.org and http://www.beebase.org.

The BLAST analysis conducted on the NCBI website found that ITS sequences from both of the selected fungal cultures possessed 100% sequence identity to ITS sequences from voucher strains of *A*. *apis*. The results of phylogenetic analysis in RAxML and MrBayes place the two fungal cultures in a single clade, along with the two voucher strains and the genome strain of *A*. *apis* (Fig 2). The ITS sequence alignment file used in these analyses along with maximum likelihood and Bayesian trees are available on Treebase (http://www.treebase.org/treebase-web/) under the accession number 16737 (http://purl.org/phylo/treebase/phylows/study/TB2:S16737).

## Discussion

The study documented, for the first time, development of fungi in the genus *Ascosphaera* in adult bumble bees. Both vegetative and reproductive stages of the fungus were detected within the body cavities of queens of three USA west coast species, *B*. *griseocollis*, *B*. *nevadensis* and *B*. *vosnesenskii*, and additionally, in workers of *B*. *nevadensis*. Until now, various species of *Ascosphaera* have been documented to develop only in bee larvae and on bee products [1, 2]. Thus, the study documents the adaptation to infect adults in one more larval bee pathogen. Species of *Aspergillus* (Ascomycota: Eurotiomycetes), classified in the same subclass of Fungi as *Ascosphaera*, cause stonebrood disease in bee larvae but have also been documented to infect adults. Although less common than chalkbrood, stonebrood has etiology similar to that of *Ascosphaera*, infecting the host via ingestion of spores [1]. The spores germinate within the gut, ultimately making mummies of larvae, similar to those produced in outbreaks of chalkbrood by *Ascosphaera*, and producing fatal infections in adults. The current study documented infections in adult bumble bees but more research is needed to determine if adults succumb to the infections.

Based on morphological and molecular analyses, this study also represents the first ever detection of the species *A*. *apis* in bumble bees. This is a new host record for *A*. *apis*. The fungus has previously been recorded only from the Asian honey bee, *Apis cerana* F., European honey bee and from the western carpenter bee *Xylocopa californica arizonensis* Cresson (Apidae) [1, 24]. The results of the BLAST analysis found 100% sequence similarity between the ITS regions of the two strains of *A*. *apis* isolated from this study, and previously sequenced strains of *A*. *apis*. These results are not surprising given that previous research has also found 100% sequence identity of this region between numerous strains of *A*. *apis* isolated from European honey bee larvae collected from several continents [6]. However, high sequence similarity at the ITS region alone does not indicate genetic homogeneity within populations of this pathogen. Twenty five variable microsatellite loci have recently been developed for this fungus and surveys of strains found in the eastern state of Maryland, USA, alone displayed between 2 and 8 alleles for each of these loci [25]. Additionally, variable intergenic markers have been developed for identification of *A*. *apis* haplotypes and these markers have demonstrated efficacy at distinguishing strains that share high similarity at the ITS region [26].

Future studies are needed to determine whether the strain of *A*. *apis* identified in this study has undergone genotypic mutations giving it the potential to infect adult insects. It is also possible that the novel ability to parasitize adult insects might be associated with epigenetic factors. Comparison of the transcriptome of the *A*. *apis* strain found in adults in the current study to those of strains affecting larvae might yield valuable insight into the pathogenicity of this fungus. The results of such a study might also broaden insights into the mechanisms that enable host shifts in insect pathogens.

The current study provides documentation of a strain of *A*. *apis* in which the hyphae do not penetrate the cuticle, and in which fruiting bodies are formed internally within the host. This form of development has previously been documented only in *A*. *aggregata*. In *A*. *apis*, 11 enzymes have been identified that assist the pathogen in penetrating the peritrophic membrane of the larval mid gut and then invading larval tissues and emerging from the host upon death of the host [27]. Further research is needed to determine whether the adults spread that pathogen and, if so, how. Perhaps the below-surface development observed in the current study is due to absence of appropriate enzymes required for breakdown of an adult cuticle. The absence of external symptoms of the fungus may be the reason that its presence has remained undetected in adult bumble bees prior to the current study, or in any other adult bee.

The source of *Ascosphaera* infection in adult bumble bees in the current study is unknown. *Ascosphaera* is common in the environment [8] wild bees could have been infected prior to capture for rearing. However, bumble bee queens that were examined after they had died had been in captivity for 21–121 days which suggests that *A*. *apis* infection was likely a mass rearing artifact that originated via contaminated pollen that was fed to the bees. This has been documented to occur in other bee species [28]. Tiny (< 2 μm) spores, believed to be *A*. *apis*, were observed within pollen patties as well as raw pollen. Although attempts were made to grow the fungus from both samples, only the pollen provisions produced mycelia. Commercial bee-feed often contains fungicides, which might explain the suppressed sporulation.

Pathogens can be spread between bee species via pollen not just during rearing but also during foraging with far greater implications [29]. Recent studies have found that cross infection of pathogens between species occurs more frequently than previously believed [30, 31]. Thus far, while *A*. *apis* causes chalkbrood diseases in larval European honey bees, the colonies are rarely treated for fungal suppression. However, if the new strain of *A*. *apis* detected in the current study is pathogenic, its spread to other native pollinators could have devastating effects.

In the current study, the fungus was detected in one *B*. *nevadensis* queen that had been frozen alive but 11 other infected queens were examined after they died. While *A*. *apis* could have been the cause of death in these queens, it is also possible that, as a facultative saprotroph, the fungus may have been colonizing immunocompromised bee tissue. Pathologists typically use Koch’s postulates to demonstrate linkage between disease symptoms and a pathogenic organism. For conducting such a study, control bees lacking *Ascosphaera* spores are needed. Since wild bees may harbor *Ascosphaera*, sterile commercial bees would be required for such a study. Currently, the only sterile commercial bee that is available in the US is *B*. *impatiens* Cresson which cannot be introduced to Oregon as it is not native to the state [32]. Hence, a trial with sterile colonies was unfeasible in the current study. In place of this, a small scale non-controlled trial was conducted in which wild bumble bee queens were inoculated with *A*. *apis* isolated in this study. Out of 50 queens that were fed pollen mixed with ascospores harvested from cultures isolated in this study, two queens that died were observed to be infected with the fungus. No living individuals in the trial contained mycelia. In the absence of a control, these data are inconclusive and further studies are needed for confirming if *A*. *apis* can cause fatal infections in adult bumble bees. Further studies are also needed to determine if *A*. *apis* affects adult bumble bees in other ways. Two colonies whose queens were infected with *A*. *apis* went directly to male production without typical prior production of new queens. This behavior may be a symptom of stress to the colony [33] caused by the presence of the fungus though other factors could also be involved.

Pathogens acquired by a species during commercial rearing also pose a threat to native counterparts [34, 35, 36, 37, 38]. In the late 1990’s, soon after Midwest-reared commercial colonies of the western US species *B*. *occidentalis* Greene were used for crop pollination in west coast states, wild counterparts declined rapidly in the region. It is speculated that, during commercial rearing, colonies were exposed to pathogens to which *B*. *occidentalis* was susceptible while the eastern US *B*. *impatiens* which was reared at the same facility, was unaffected. Pathogen spillover from infected commercial colonies released in the west is believed to have led to the observed rapid decline of *B*. *occidentalis* in the region [34, 35, 36, 37]. The potential for pathogen spillover is highlighted in the study by Murray et al. [38] that documented the presence of infected ‘reservoir’ populations of bumble bees in regions adjacent to greenhouses where commercial colonies were released.

The detection of a potential new strain of *A*. *apis* in adult bumble bees adds to pollination concerns in western USA, an important agricultural region where many bee pollinated crops are raised for production of nuts, fruits, vegetables, or for seed [39]. While many of these crops are pollinated by managed European honey bees, declines due to the Colony Collapse Disorder, Varroa mites and other factors have reduced the availability of hives and increased the cost of rentals [40, 41, 42]. European honey bee hive losses have drawn global attention to the role of bumble bees as pollinators in natural and managed ecosystems. Worldwide, there are 250 bumble bee species [43] but wild populations are not always synchronized with bloom or inadequate in abundance for crop pollination [44]. In addition, declines in native bumble bee populations have been reported due to habitat fragmentation, pesticide exposure, and diseases [45, 46, 47, 48, 49]. The demand for managed colonies of bumble bees for crop pollination has thus increased, and this has spurred interest in mass production of western bumble bee species by large and small local businesses. These include the three species examined in the current study. *Bombus vosnesenskii* is distributed primarily along coastal regions in western USA but is the dominant species in the agriculturally rich Willamette Valley in western Oregon. *Bombus nevadensis* and *B*. *griseocollis* are less abundant in the region but have wider distributions across the US [50, 51]. Currently, for colony initiation, wild queens of western native species are collected annually in spring and placed in rearing boxes for colony initiation. If *A*. *apis* acquired during rearing is pathogenic to bumble bees, western bumble bee species are at risk. Thus, further research is critically needed to determine potential risks associated with *Ascosphaera* infections in bumble bee colonies to other bumble bee species reared in captivity and to native populations.

In summary, the current report on the detection of the fungus *Ascosphaera* in adult bumble bees represents a new development-stage and host record for the fungus which has previously been recorded only from larval stages of bees. The new hosts belong to three species of bumble bees being considered for commercial colony production. Different species of *Ascosphaera* cause chalkboard diseases in larvae of solitary bees and honey bees but the genus has never been reported earlier in any adult bee. Also, the species *A*. *apis* detected in the study has been reported earlier only from larvae of Asian and European honey bees, and a carpenter bee species. The fungal infection observed in the current study likely occurred via ingestion of contaminated European honey bee pollen that was provided to the bumble bees while they were reared indoors. More research is needed to determine if the fungus can cause fatal infections in adults. Rapid decline of a key western bumble bee pollinator has led to speculations that pathogens acquired during rearing can have spillover effects in nature when infected bees are used for commercial crop production. Pathogens can also be transferred between bee pollinator species via contaminated pollen both during rearing of bees and while foraging on flowers. Thus, the discovery of *Ascosphaera* in adult bumble bees documented in the current study has important implications for commercial production of bumble bee colonies and highlights potential risks to native bees via pathogen spillover from infected bees and infected pollen.
